# Getting Into the Goldilocks Zone: Finding the Right Nutrient Balance in Saudi Prison Food

**DOI:** 10.7759/cureus.48545

**Published:** 2023-11-09

**Authors:** Aroub Alnasser

**Affiliations:** 1 Food Science and Nutrition, King Saud University, Riyadh, SAU

**Keywords:** plant-based, dietary reference intake, saudi arabia, nutritional analysis, prison menu

## Abstract

Background

The general characteristics of prison menus worldwide can include unhealthy, low-quality options. This imbalance can lead to poor health consequences. In seeking the optimal “Goldilocks Zone” of nutritional adequacy, this study analyzed the uniform seven-day cycle menu for both Saudi female and male prisons and compared it to standard dietary recommendations.

Aim

The United Nations recognizes that the “food security and nutritional status of the most vulnerable” are projected to “deteriorate further due to the health and socio-economic impacts of the COVID-19 pandemic, according to the State of Food Security and Nutrition in the World 2021 report. This study focuses on the nutritional status of female and male Saudi prisoners and is designed to understand and quantify the prevailing levels of nutrient provision to Saudi prisoners based on a seven-day cycle menu.

Methods

A cross-sectional, descriptive study design was utilized to examine the seven-day cycle national menu, which is consistently applied to both male and female Saudi prisoners. We calculated the nutrient provision from this menu using data on the typical nutrient content of the provided food. Subsequently, we compared the determined levels of nutrients with the reference Dietary Reference Intakes (DRIs).

Results

For most nutrients, the levels provided in the menu were adequate. Prisoners receive an adequate supply of energy and macronutrients, as well as sufficient quantities of most minerals and vitamins. The fiber content of the diet was notably low, and the sodium content was above the recommended upper limits. Mineral and vitamin levels were low for potassium and Vitamin A and slightly below the recommended level for calcium. The potential health implications of long-term nutrient imbalances are discussed, along with suggestions for addressing these imbalances by introducing different foods.

Conclusion

The Saudi prison menu operates within the Goldilocks Zone of nutritional balancing. However, optimizing the standard prison menu to more closely meet nutritional and prisoner health goals and to offer more plant-based options for prisoners to help address the United Nations - Sustainable Development Goals and Saudi Vision 2030 is worthy of further discussion and research.

## Introduction

Access to nutritious foods and nutrient-balanced meals is a fundamental characteristic of a healthy life and human development. Good nutrition is essential not only for the well-being of the individual but also for society as a whole. Nutrition has been a critical part of human advancement, with research always finding new information on how best to meet nutritional needs. Despite this, nutrient imbalances still exist in global diets [1]. Prison menus often reflect this imbalance, with the WHO finding that many prisons globally do not provide adequate nutritional options necessary to ensure basic human needs are met [2]. The WHO recognizes that “admission to prison can be the first time (prisoners) they have had a settled life with adequate nutrition and a chance to reduce their vulnerability to ill health and social failure. Further, WHO exhorts prison administrations “to ensure that prisoners have access to a nutritionally adequate and balanced diet” and “to ensure that the options are healthy.” In terms of the nutritional approach, WHO also recommends that prisons “provide guidance to prisoners on the nutritional content of the food provided” [2]. Provision of nutritional adequacy and energy intake is critical to the health of prisoners and their development, both during and after a prisoner’s sentence. After release, the health issues of prisoners become the health concerns of the general community in the long term [3]. Prisoners may also come from poorer backgrounds or geographic areas, meaning that their time in prison might be their only opportunity to eat three balanced meals a day and make healthier nutritional choices [2]. Consequently, prison can be an opportunity for education on nutrition and dietary requirements, leading to routines and dietary habits maintained after release. There is a general lack of studies on the nutritional adequacy of prison menus around the world [4]. Most studies have been conducted in the United States of America, the United Kingdom, or other Western European prisons [5]. This is often due to limited access to prisons and the reluctance of inmates to engage in such studies in other parts of the world [6-8]. A literature review has revealed a dearth of studies on this topic within Saudi Arabia. The main aim of this work is to establish which, if any, disparities exist between the nutrition and energy balance provided by a typical Saudi prison menu and that of the ideal balance in the context of an optimized “Goldilocks Zone” of nutrition and to consider how nutritional analysis and profiling may also serve the Sustainable Development Goals (SDGs) of the United Nations and Saudi Arabia’s Vision 2030 [9]. Furthermore, and in context, the Kingdom of Saudi Arabia is experiencing enormous directional change focusing on sustainability, innovation, and the future as part of the Vision 2030 initiative. Within the expansive national scope of the program is the goal of building “flexible and sustainable food systems at the local and international levels that ensure the availability of healthy and safe food for all segments of society, and to ensure the availability of all possible tools and measures that help achieve the goals of sustainable development in 2030” [10]. Analyzing the nutrient content of a prison menu used for male and female prisoners is a part of this conversation and work. The results of this analysis can serve not only to inform but also help guide Vision 2030 towards its noble goal of ensuring “healthy and safe food for all segments of society” [10].

## Materials and methods

A cross-sectional descriptive study design was used to study the seven-day cycle national menu uniformly applied to all Saudi prisons. The prison menu used for the study was a weekly cycle menu developed by the Nutrition Administration for the General Directorate of Prisons in cooperation with the Prison Nutrition Committee. The menu includes breakfast, lunch, and dinner for male and female prisoners throughout Saudi Arabia. A menu outline is provided in Tables 1-3 (translated to English for this study), including allocated quantities.

**Table 1:** Saudi prison breakfast menu weekly cycle. *The work-week begins on Sunday in Saudi Arabia.

Day	Item (weight)
Sunday*	Bread (100g), Pastry (60g), Boiled egg (100g), Dates (100g), Juice (250ml), Tea (2g), Drink water (330ml)
Monday	Bread (100g), Fava bean dip (70g), Olives (30g), White cheese (40g), Tea (2g), Drink water (330ml)
Tuesday	Bread (100g), Lentil Dip (70g), Triangle cheese (30g), Jam (30g), Milk (200ml), Tea (2g), Drink water (330ml)
Wednesday	Bread (100g), Fava bean dip (70g), White cheese (40g), Dates (100g), Tea (2g), Drink water (330ml)
Thursday	Bread (100g), Lentil Dip (70g), Triangle cheese (30g), Honey (30g), Milk (200ml), Tea (2g), Drink water (330ml)
Friday	Bread (100g), Pastry (60g), Boiled egg (100g), Dates (100g), Juice (250ml), Tea (2g), Drink water (330ml)
Saturday	Bread (100g), Lentil Dip (70g), Tahini halva (60g), White cheese (40g), Tea (2g), Drink water (330ml)

**Table 2:** Saudi prison lunch menu weekly cycle.

Day	Item (weight)
Sunday	Bread (100g), Rice (170g), Frozen chicken (250g), Potato & zucchini stew (100g), Salad (150g), Fruit (150g), Tea (2g), Drink water (330ml)
Monday	Bread (100g), Rice (170g), Frozen lamb meat (250g), Frozen Mulukhiyah (80g), Salad (150g), Fruit (150g), Tea (2g), Drink water (330ml)
Tuesday	Bread (100g), Rice (170g), Frozen Fish (350g), Salad (95g), Fruit (150g), Tea (2g), Drink water (330ml)
Wednesday	Bread (100g), Rice (170g), Frozen chicken (250g), Mixed fresh vegetables (100g), Salad (150g), Laban (200ml), Tea (2g), Drink water (330ml)
Thursday	Bread (100g), Rice (170g), Lamb meat (250g), Frozen vegetables stew (100g), Salad (150g), Fruit (150g), Tea (2g), Drink water (330ml)
Friday	Bread (100g), Rice (170g), Chicken (250g), Frozen Mulukhiyah (80g), Salad (150g), Tea (2g), Drink water (330ml)
Saturday	Bread (100g), Rice (170g), Lamb meat (250g), Salad (150g), Fresh vegetables (100g), Fruit (150g), Tea (2g), Drink water (330ml)

**Table 3:** Saudi prison dinner menu weekly cycle.

Day	Item (weight)
Sunday	Bread (100g), Lentil dip (70g), Triangle cheese (30g), Honey (30g), Tea (2g), Drink water (330ml)
Monday	Bread (100g), Burger (100g), Cheese slice (40g), Ketchup (18g), Juice (250ml), Tea (2g), Drink water (330ml)
Tuesday	Bread (100g), White cheese (40g), Tahini halva (60g), Olives (30g), Yogurt (170g), Tea (2g), Drink water (330ml)
Wednesday	Bread (100g), Beef kebab (100g), Roasted Potatoes/ French fries (100g), Ketchup (18g), Fruit (150g), Tea (2g), Drink water (330ml)
Thursday	Bread (100g), Potato & zucchini stew (100g), Chicken (50g), Yogurt (170g), Tea (2g), Drink water (330ml)
Friday	Bread (100g), Fava bean dip (70g), Triangle cheese (30g), Jam (30g), Fruit (150g), Tea (2g), Drink water (330ml)
Saturday	Bread (100g), Mixed fresh vegetables (100g), Chicken (50g), Laban (200ml), Tea (2g), Drink water (330ml)

Due to the absence of a Saudi nutrient database software [11], this study used several food composition tables to manually calculate the nutrient content of the typical standard prison meal (male and female). The food composition tables pertained primarily to foods found in Bahrain [12], Saudi [13], and Kuwait [14]. For food items unavailable in these three countries, food composition values were taken from data in the UK [15] and US food databases [16]. The analysis did not include food that prisoners might purchase from a prison commissary or that visitors might bring in. Included in the analysis were daily caloric intake (energy, kcal) and the percentage distribution of macronutrients (protein, fat, and carbohydrates). The amount of fiber, minerals, and vitamins was also calculated. The vitamins were retinol (vitamin A), thiamin, riboflavin, niacin, and vitamin C. The minerals were calcium (Ca), potassium (K), phosphorus (P), and iron (Fe). The amount of sodium (Na) in the diet was also calculated. Data from the DRI was used to establish the energy and protein values in the menu [17]. The DRI uses values for height and weight to inform a comprehensive evaluation of nutritional adequacy for an individual. In this work, values for height and weight were assumed to be the average height and weight of Saudi Arabian residents. The average male height in Saudi Arabia is 1.70 m with a weight of 80.9 kg, whilst the average height for women is 1.58 m with a weight of 73.6 kg [18]. Furthermore, calculations were made assuming a prisoner age of 36, as this is the mean prisoner age globally [19]. Physical activity level was assumed to be sedentary. Macronutrients as a proportion of energy and micronutrients were compared with DRIs for all nutrients except protein, where the Accepted Macronutrient Distribution Range (AMDR) was used. The weekly menu was disaggregated into food groups according to the Saudi Healthy Palm food group guidelines (i.e., cereal, protein, milk, vegetables, and fruits) using Microsoft Excel software [20]. The statistical analysis was carried out using Microsoft Excel 2020. Additionally, an independent rater reviewed a random sample, two days from the seven-day cycle menu, of the food analysis sheets. Taking the number of identically scored items from the task analysis sheet and dividing it by the total number of items, then multiplying the quotient by 100%, the measure of interrater agreement was calculated as 97%. A slight discrepancy between the rater and this study’s findings was noted. Neither human nor animal subjects were used in this study. Therefore, no ethics approval was required.

## Results

Analysis of food groups

According to the prison menu, a typical breakfast consisted of bread, bean dip, and milk or juice. Lunch mainly included rice, fruit, salad, vegetable stew, and meats such as chicken/fish. Various types of meat dishes and dairy products or juice were served for dinner. A comparison made of the prison menu foods against the Healthy Food Palm food group guidelines showed that there were minimal portions of fruits, vegetables, and dairy. It revealed that cereal and bread, as well as meat and substitutes, were overrepresented and exceeded recommendations (Table 4).

**Table 4:** Comparison of Saudi prison menu food groups with the Healthy Palm food groups. *Healthy Food Palm Recommended Servings

Food Group	Prison menu	The Saudi Healthy Food Palm*
Cereal and bread	15	6-11
Meat and substitutes	4	2-3
Vegetables	3	3-5
Fruit	2	2-4
Milk and dairy	2	2-4

Energy, macronutrients, and micronutrients

The daily calorific intake based on the foods present in the menu analyzed revealed that the supplied energy amount was 2654±108 kcal. The percentage distribution of macronutrients was 55% carbohydrates, 18% protein, and 28% fat. Total dietary fiber was an average of 18±3g per day. Vitamin and mineral levels are presented in Table 5.

**Table 5:** Micronutrient content of Saudi prison menu and its relationships to DRI. Data has been represented as mean (SD), and %. %: Percentage, DRI: Dietary Reference Intakes, mg: milligram, µg: microgram.

Micronutrient		Unit	Mean ± SD	DRI (males)	% DRI	DRI (females)	% DRI
Sodium	mg		2467±60	1500	164	1500	164
Potassium	mg		1592±32	3400	47	2600	61
Calcium	mg		856±15	1000	86	1000	86
Phosphorus	mg		1668±24	700	238	700	238
Iron	mg		18±5	8	225	18	100
Retinol	µg		326±85	900	36	700	47
Thiamin	mg		2±1	1.2	166	1.1	182
Riboflavin	mg		1±0	1.3	77	1.1	91
Niacin	mg		19±6	16	119	14	136
Vitamin C	mg		82±53	90	91	75	109

## Discussion

This study analyzed the nutritional adequacy of a seven-day prison cycle menu in the Kingdom of Saudi Arabia using a one-week menu sample. The menu provided suggested that cereal and bread, as well as meat and substitute food group categories, have a high prevalence in the diets of Saudi prisoners. Rice with a meat-based meal was found to be the most common food served for lunch, and pitta bread was provided with each meal three times a day. On average, the mean daily calorific intake of prisoners, according to the prison menu, was 2645 kcal. The Saudi prison menu, therefore, provides adequate caloric content, ensuring that the most basic nutritional needs of all inmates are met. As with prison menus worldwide, the Saudi prison diet does not differentiate between male and female prisoners [21,22]. However, nutritional needs differ between males and females. According to DRIs, the daily caloric intake for prisoners was 14.4% higher for males and 36.1% higher for females than the recommended intake levels. This intake was also 288 kcal higher than the daily caloric intake of the general Saudi population. On a positive note, female inmates receive adequate levels of iron. However, they consume too many calories, leading to increased BMI and weight gain. Work elsewhere suggests that a daily caloric intake above recommended levels is characteristic of diets within the general population of Saudi Arabia, not just prison menus, especially for women [23]. Studies into other segments of the Saudi population show that the average daily caloric intake of 2,357 kcal is close to the DRI for men (2,313 kcal) but is 21.2% higher than that for women (1,944 kcal). However, it is crucial to recognize that the reduced physical activity of female prisoners, compared to females in the general population, makes the issue of excessive calorie intake more pronounced in prisons. The daily caloric intake of Saudi prisoners is comparable to that of prisoners in other parts of the world. A study on the nutrient balance of prison menus in Poland [24] found that daily caloric intake was 2708 kcal, indicating an intake level similar to that of the KSA. The issue of excessive calorie intake is more pronounced in countries such as Canada (+22% and +45% of the DRI for men and women, respectively) [25]. In less developed economies such as Ghana [26], the caloric intake is nearly 9% lower than the DRI for men and 9% higher for women. The percentage distribution of macronutrients in the Saudi prison menu corresponded well with DRIs and the AMDR for protein. The percentage of carbohydrates was 55%, which is the mean of the recommended range of 55-65%. The percentage of protein (18%) was also within the recommended range (10-35%) but was towards the lower end of the range. The percentage distribution of fats was 28%, which was near the mean of the recommended range of 20-35%. The Saudi prison menu provides an adequate percentage distribution of three main macronutrients (carbohydrates, protein, and fats) based on the DRIs for carbohydrates and fats and AMDR for protein. Within the global context, the provision of macronutrients by the Saudi prison menu performed better than in Poland and Ghana but not as well as in Canada and Australia [27]. The Saudi prison menu had a higher protein content and a lower carbohydrate content than those of the Polish and Ghanaian menus. The fat content of the Saudi prison menu was considerably higher than those of all other prisons.

Although the Saudi prison diet indicates that inmates consume more than the recommended intake of grains (according to the Saudi Healthy Palm), the nutritional analysis reveals that there is not enough fiber in the prison diet. Prisoners receive 18g of fiber daily, as opposed to the DRIs for men (38g) and women (25g). It was also very low compared to the provision of fiber in the prison menus of all other countries except for the USA. A practical way of providing more fiber to Saudi prisoners could be to replace some of the bread provided with each meal with wholegrain bread. Disparities between the levels of vitamins and minerals in the Saudi prison diet and the DRIs were noted for potassium, Vitamin A, and calcium. However, this issue is not unique to Saudi prisons; such shortfalls are commonly observed in prison food menus worldwide. Inadequacy in the provision of potassium is demonstrated in prison menus of Ghana (31% of DRI), the USA (62% of DRI), Poland (98% of DRI), and Australia (94% of DRI). Likewise, an inadequacy in providing calcium is also present in Ghana (93% of DRI for men) and Poland (44% of DRI). Provision of vitamin A is also below the DRI in Ghana (35% and 44% of DRI for men and women, respectively). Sodium, phosphorus, and iron were all almost double the DRI in the Saudi prison diet. The highest mineral intake was sodium, which amounted to 2509 mg or 167% of the recommended 1,500 mg, and was also higher than the recommended maximum intake limit of 2,300 mg. However, the issue of excess sodium in the Saudi prison menu is not as pronounced as that of prison menus from around the world. For example, the sodium content of the Polish prison menu is more than 3x the DRI. Other studies have found the sodium content of Polish prison menus to be as much as 5x the DRI [28]. Excessive sodium intake is also an issue within the greater Saudi population, predominantly due to unexpected sources of salt, such as vegetables and salad [29]. Dishes like mulukhiyah and salad, which are available fresh year-round and are naturally low in sodium, are already on the Saudi prison menu. The challenge is to remove sodium from the preservation and cooking processes as well as to address the issue of how prisoners are seasoning their foods. Given the inadequacies of certain minerals and vitamins in the Saudi prison menu diet, it is essential to explore how these nutrient imbalances can be addressed. There are many ways of introducing these minerals and vitamins into the prison menu diet. However, any adjustments need to take into account three factors. Firstly, the menu adjustments need to be made in a way that ideally reduces rather than increases sodium. At the same time, the adjustments would reduce fat content in the diet and increase fiber, potassium, vitamin A, and calcium. Lastly, adjustments made need to be realistic in the context of cost constraints. One potentially promising means of rebalancing the Saudi prison menu nutrient levels whilst keeping costs down is plant-based nutrition. These protein options contain high levels of fiber without the high-fat content that is often associated with high-protein foods like red meat [30]. However, the provision of minerals and vitamins from plant-based proteins varies depending on the plant source. For instance, a recent study demonstrated that a 100-gram serving of plant-based protein paste provides 8% of the daily potassium requirement for women [30]. Other studies have shown that plant-based protein foods derived from peas have high levels of calcium and low levels of sodium [30]. As well as providing health benefits for its consumers, plant-based proteins also offer opportunities that help to realize the UN SDGs and Saudi Vision 2030 [9] through reduced environmental impact. Replacing meat options with fiber-rich, plant-based proteins could theoretically reduce costs, reduce environmental impact and deliver improved prisoner health outcomes through novel diet menu options. While this study offers valuable insight into the nutritional content of the Saudi prison diet, the findings come with caveats. While the data offers an analysis of the Saudi prison cycle menu, there is no data or analysis of prison food consumption, nor did this analysis include a breakdown of foods purchased at the commissary or brought in for prisoners by visitors. Further, this study did not analyze prisoner BMI, weight change, energy levels, sleep cycles, physical activity, the presence of disease, or other factors.

## Conclusions

The nutrient analysis of the seven-day cycle menu for male and female prisoners in Saudi Arabia carried out in this work found that prisoners receive adequate energy intake. However, this energy intake may be too high for women. Prisoners receive a good balance of macronutrients, though they would benefit from a reduction in fats. There is also evidence for the need to increase dietary fiber and potassium, both of which could be addressed by introducing more plant-based, nutrient-dense foods. The provision of minerals and vitamins is also generally good, though sodium content should be reduced and the provision of potassium, calcium, and vitamin A increased. The introduction of more plant-based options, alongside more low-sodium, nutrient-dense traditional foods, can be explored as a means of helping administrators and health professionals realize an optimized "Goldilocks Zone" of nutrition for Saudi prisoners. These efforts may, as well, complement and support the UN SDGs and Saudi Vision 2030 to promote the "good health and wellbeing" of the people and the planet.

## References

[1] Passarelli S, Free CM, Allen LH (2022). Estimating national and subnational nutrient intake distributions of global diets. Am J Clin Nutr.

[2] Yordi Aguirre I, Ahalt C, Atabay T (2014). Prisons and Health. World Health Organization.

[3] Australian Institute of Health and Welfare: Health of prisoners (2021). Australian Institute of Health and Welfare: Health of prisoners. https://www.aihw.gov.au/reports/australias-health/health-of-prisoners.

[4] Lyons H (2012). Food, Farming, and Freedom : Promoting a Sustainable Model of Food Justice in America’ s Prisons. Food, Farming, and Freedom : Promoting a Sustainable Model of Food Justice in America’ s Prisons FOOD, FARMING, AND FREEDOM Promoting a Sustainable Model.

[5] Smoyer AB (2019). Food in correctional facilities: a scoping review. Appetite.

[6] Simanovic T, Gosev M (2019). Is food more than a means of survival? An overview of the Balkan prison systems. Appetite.

[7] Edwards JA, Hartwell HJ, Reeve WG, Schafheitle J (2007). The diet of prisoners in England. British Food J.

[8] Bukhari R, Al-Sulaimi A, Fadaak A, Balhaddad A, AlKhalfan A, Tantawi M El, Al-Ansari A (2017). Oral health amongst male inmates in Saudi prisons compared with that of a sample of the general male population. South Afr Dental J.

[9] (2021). Saudi Vision 2030. https://www.vision2030.gov.sa/.

[10] (2022). Saudi Press Agency: Eng. Al-Fadhli: Saudi Arabia has taken important steps to build effective and flexible food systems. https://www.spa.gov.sa/en.

[11] Naaman RK, Almasaudi A, Albajri E, Naseeb M (2023). Current use of food composition database and dietary analysis software in Saudi Arabia: a review study. J Food Composition Anal.

[12] Musaiger AO (2021). Food composition tables for Kingdom of Bahrain. https://www.cabdirect.org/cabdirect/abstract/20113393010.

[13] Musaiger AO (2006). Food Composition Tables for Arab Gulf Countries (Gulfoods)” Arab Center for Nutrition, Manama-Bahrain.

[14] Dashti BH, Al-Awadi F, Khalafawi MS, Al-Zenki S, Sawaya W (2001). Nutrient contents of some traditional Kuwaiti dishes: proximate composition, and phytate content. Food Chem.

[15] McCance RA, Elsie Widdowson (2021). McCance and Widdowson’s the Composition of Foods. Royal Society of Chemistry.

[16] (2021). US Department of Agriculture ARService‏: FoodData Central. https://fdc.nal.usda.gov/..

[17] Institute of Medicine (2000). DRI Dietary Reference Intakes: Applications in Dietary Assessment.

[18] (2021). WorldData: Average height of men and women worldwide. https://www.worlddata.info/average-bodyheight.php.

[19] (2021). Federal Bureau of Prisons: BOP Statistics: Average Inmate Age. https://www.bop.gov/about/statistics/statistics_inmate_age.jsp.

[20] Al-Dkheel M (2021). Al-Dkheel M: Dietary Guidelines for Saudis. https://www.moh.gov.sa/en/Ministry/MediaCenter/Publications/Documents/finalenglish.

[21] Cook EA, Lee YM, White BD, Gropper SS (2015). The diet of inmates: an analysis of a 28-day cycle menu used in a large county jail in the State of Georgia. J Correct Health Care.

[22] Herbert K, Plugge E, Foster C, Doll H (2012). Prevalence of risk factors for non-communicable diseases in prison populations worldwide: a systematic review. Lancet.

[23] Alfadhli EM (2016). Macronutrients imbalance and micronutrient deficiencies among healthy Saudi physicians in Al Madina, Saudi Arabia. Saudi J Med Med Sci.

[24] Kosendiak A, Stanikowski P, Domagała D, Gustaw W (2020). Gluten-free diet in prisons in Poland: nutrient contents and implementation of dietary reference intake standards. Nutrients.

[25] Johnson C, Labbé C, Lachance A, LeBlanc CP (2022). The menu served in Canadian penitentiaries: a nutritional analysis. Nutrients.

[26] Ama N, Agyapong F, Annan RA, Apprey C (2018). Assessment of food and nutrient provision within prisons in the Ashanti Region of Ghana. Asian Food Sci J.

[27] Hannan-Jones M, Capra S (2016). What do prisoners eat? Nutrient intakes and food practices in a high-secure prison. Br J Nutr.

[28] Stanikowski P, Michalak-Majewska M, Domagała D, Jabłońska-Ryś E, Sławińska A (2020). Implementation of dietary reference intake standards in prison menus in Poland. Nutrients.

[29] Alkhalaf MM (2017). Nutrition and body composition as risk factors of non-communicable diseases in Saudi Arabia. https://theses.gla.ac.uk/8533/1/2017AlkhalafPhD.pdf.

[30] Švarc PL, Jensen MB, Langwagen M, Poulsen A, Trolle E, Jakobsen J (2022). Nutrient content in plant-based protein products intended for food composition databases. J Food Comp Anal.

